# Station of the fetal head at complete cervical dilation impacts duration of second stage of labor

**DOI:** 10.1016/j.eurox.2019.100100

**Published:** 2019-10-31

**Authors:** Elisabeth Ludvigsen, Finn Egil Skjeldestad

**Affiliations:** a Division of Surgery, Oncology and Women’s Health, University Hospital of North Norway, Norway; b Research Group Epidemiology of Chronic Diseases, Institute of Community Medicine, UiT the Arctic University of Norway, Tromsø, Norway

## Abstract

**Objective:**

To examine the association between station of the fetal head at complete cervical dilation and duration of second stage of labor, as well as prolonged second stage of labor, without and with the use of analgesia (EA).

**Study design:**

We conducted a population-based retrospective cohort study of 3311 women with a singleton pregnancy, gestational age ≥ 37^0^ weeks, and cephalic presentation. Station of the fetal head at complete cervical dilation was categorized as at the pelvic floor, beneath the ischial spines, but above the pelvic floor, and at or above the ischial spines. In logistic regression analysis, we defined prolonged second stage of labor as > 2 h without and > 3 h with EA in nulliparous women, and > 1 h and > 2 h, respectively, in parous women.

**Results:**

Survival curves demonstrated longer durations of second stage of labor in nulliparous women and women with EA in each category of station of fetal head. The adjusted odds ratio of prolonged second stage of labor was 13.1 (95% confidence interval (CI): 8.5-20.1) times higher when the fetal head was beneath the ischial spines, but above the pelvic floor, and 32.9 (95% CI: 21.5-50.2) times higher when the fetal head was at or above the ischial spines compared to at the pelvic floor.

**Conclusion:**

Station of the fetal head at complete cervical dilation was significantly associated with duration of second stage of labor.

## Introduction

The second stage of labor encompasses the events between complete cervical dilation and delivery of the fetus. Management of this second stage is commonly based on its duration.

Studies [[1], [2], [3]] have reported inconsistent results on the impact of epidural analgesia (EA) on duration of first stage of labor. However, most studies [1,2,4,5] agree that EA can increase duration of second stage. In 2003 the American College of Obstetricians and Gynecologists (ACOG) ^6^ defined prolonged second stage of labor as > 2 h without and > 3 h with EA in nulliparous women, and > 1 hour without and > 2 h with EA in parous women. The optimal duration and management of the second stage of labor is still being debated. [7,8] Zhang et al [[9], [10], [11]] challenged existing knowledge by stating a longer duration of first and second stage of labor than has been previously accepted. They found the 95^th^ % of duration of second stage of labor to be 3.6 and 2.8 h in nulliparous women with and without EA, respectively, and suggested that the 95^th^ % is more useful in the assessment of normal progression of second stage of labor. [10] These and other reports [12,13] led the ACOG/Society for Maternal- Fetal Medicine (SMFM) to publish new labor management guidelines [14], which accepted an additional hour of duration of the second stage of labor in both nulliparous and parous women before diagnosing arrest. The new accepted duration was even longer when EA is administered. However, station of the fetal head at complete cervical dilation and the rate of fetal descent are not included in the new guidelines in regard to normal duration of second stage of labor or the definitions of prolonged second stage of labor. The objective of this study was to examine the association between station of the fetal head at complete cervical dilation and duration of second stage of labor, as well as prolonged second stage of labor, without or with the use of EA.

## Materials and Methods

We conducted a population based retrospective cohort study of women who gave birth from January 1, 2011 to December 31, 2013 in the north and middle parts of Troms, Norway. Of the 4 545 women who gave birth during this period, we included 3311 women with a singleton pregnancy, gestational age ≥ 37^0^ weeks, and cephalic presentation who reached the second stage of labor, and had valid information on station of the fetal head at complete cervical dilation and duration of first and second stages of labor. Data were retrospectively transferred from the electronic medical birth record (PARTUS) into a case-report-form, which was validated manually against all source information by one of the authors (EL) and a medical student. Source information comprised the electronic medical birth record, medical notes during pregnancy and delivery (provided by midwives and obstetricians), partographs, the “antenatal fact sheet” (helsekort for gravide), the anesthesia report form and the personal health form provided by the women.

The primary outcome was duration of second stage of labor, defined as min from complete cervical dilation to expulsion of the fetus. The main exposure was station of the fetal head at complete cervical dilation (i.e.at the start of second stage of labor), which categorized in three groups; at the pelvic floor, beneath the ischial spines, but above the pelvic floor, and at or above the ischial spines. Our institution practiced a -3 to +3 scale for assessment of station of the fetal head. Cervical dilation and station of the fetal head was assessed by digital vaginal examination, which was performed at admission by the midwife or the obstetrician in charge. Regular contractions and cervical dilation > 3-4 cm defined the start of labor. For women with a cervical dilation of > 4 cm at admission, the midwife estimated the start of active labor based on information from the woman and vaginal examination. The time variable “onset of labor” was validated against available source information during data entry.

All inductions of labor were carried out at the maternity department at the University Hospital of Northern Norway. Specialist consultants assessed the indications for labor induction. Based upon cervical ripening (Bishop Score), the method of induction was either a cervical ripening agent (misoprostol 25 micrograms or when having a previous cesarean delivery, dinoproston 3 milligrams) administered vaginally or artificial rupture of membranes (when intact) followed by an oxytocin regimen administered intravenously.

It is established knowledge that duration of second stage of labor varies by parity [6,15,16]; therefore all analyses on this duration were stratified by parity. In order to compare our results with internationally accepted definitions (ACOG 2003) the outcome variable “prolonged second stage of labor” was transformed into a binary categorical variable: duration ≥ 3 h with EA and ≥ 2 h without EA for nulliparous women and duration ≥ 2 h with EA and ≥ 1 hour without EA for parous women. Further, we estimated the 50^th^, 90^th^ and 95^th^ % of duration of second stage of labor. The time variable “first stage of labor” was dichotomized based on the 90^th^ % and stratified by parity. Pre-pregnancy body mass index was defined as weight divided by the square of the body height (kg/m²) and categorized according to the World Health Organization’s classification.

Chi-square test for independence was used for categorical variables to explore the relationship between maternal and labor characteristics and station of the fetal head and prolonged second stage of labor. The Mann Whitney U test was used to examine duration of second stage of labor by parity, and the Kruskal-Wallis test was used to compare median durations of first and second stage between groups. The distribution of duration of the second stage of labor by category of station of fetal head was displayed by survival curves, censoring women with cesarean delivery. Furthermore, binary logistic regression was performed to predict the odds of having prolonged second stage of labor based on station of the fetal head, after adjusting for possible confounding factors. Parity and use of EA were included in the definition of prolonged second stage of labor, and thus we did not adjust for them. Oxytocin was considered a mediating factor and therefore was not included in the analyses. Statistical analyses were performed using IBM SPSS statistics version 24.0. P-value < 5 % was considered statistically significant.

The Regional Committee for Medical and Health Research Ethics (REC North 2013/1208) and the Patient Ombudsman, University Hospital of North Norway, Tromsø, approved the study protocol.

## Results

Of the 3311 women included in the analysis 42 % were nulliparous and 58 % were parous. The maternal characteristics age and parity; and the labor characteristics gestational age, onset of labor, use of EA, prolonged first stage of labor and fetal birth weight differed significantly by categories of station of the fetal head at complete cervical dilation, whereas pre-pregnancy body mass index and onset of labor did not (Table 1).

**Table 1:** Maternal and labor characteristics by station of the fetal head at complete cervical dilation.

Variables	At pelvic floor n = 1553 (%)	Beneath ischial spines n = 985 (%)	At/above ischial spines N = 773 (%)	P-value
Maternal age (years) < 25 25-34 ≥ 35	254 (16.4) 975 (62.8) 324 (20.9)	214 (21.7) 566 (57.5) 205 (20.8)	140 (18.1) 473 (61.2) 160 (20.7)	.02
Parity Nulliparous Parous	453 (29.2) 1100 (70.8)	536 (54.4) 449 (45.6)	397 (51.4) 773 (48.6)	< .01
Pre-pregnancy BMI (kg/m^2^) < 18,5 18,5 -24,9 25,0-29,9 > 30,0 Missing	179 (11.5) 884 (56.9) 304 (19.6) 174 (11.2) 12 (0.8)	107 (10.9) 560 (56.9) 190 (19.3) 113 (11.5) 15 (1.5)	60 (7.8) 433 (56.0) 166 (21.5) 102 (13.2) 12 (1.6)	.07
Gestational age (weeks) 37- 38 39- 40 41- 42	226 (14.6) 889 (57.2) 438 (28.2)	140 (14.2) 524 (53.2) 321 (32.6)	85 (11.0) 395 (51.1) 293 (37.9)	< .01
Induction of labor	324 (20.9)	205 (20.8)	163(21.1)	.99
Prolonged 1^st^ stage of labor^1^	106 (6.8)	109 (11.1)	120 (15.5)	< .01
Oxytocin in 1^st^ stage	180 (11.6)	183 (18.6)	167 (21.6)	< .01
EA in 1^st^ stage	188 (12.1)	225 (22.8)	249 (32.2)	< .01
Fetal birthweight > 4000 g	275 (17.7)	149 (15.1)	185 (23.9)	< .01

P-values from Chi-square tests.

^1^Duration >90^th^ %.

BMI, body mass index; EA, epidural analgesia.

**Fig. 1:**
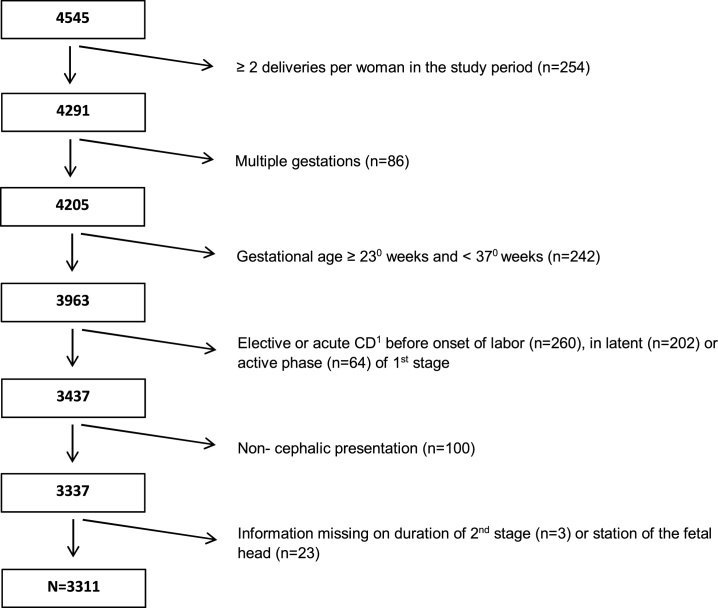
Flowchart - selection of study population.

EA was administered to 32.3 % of nulliparous and 11.1 % of parous women during the first stage of labor. Median duration of the first stage of labor was 249 min in nulliparous and 150 min in parous women not receiving EA, and this duration nearly doubled in both nulliparous and parous women receiving EA. The station of the fetal head at complete cervical dilation was diagnosed at/above the ischial spines in 37.1 % of nulliparous and 38.8 % of parous women who received EA versus 24.6 % and 17.1 %, respectively, among women who did not (Fig. 1).

**Fig. 2:**
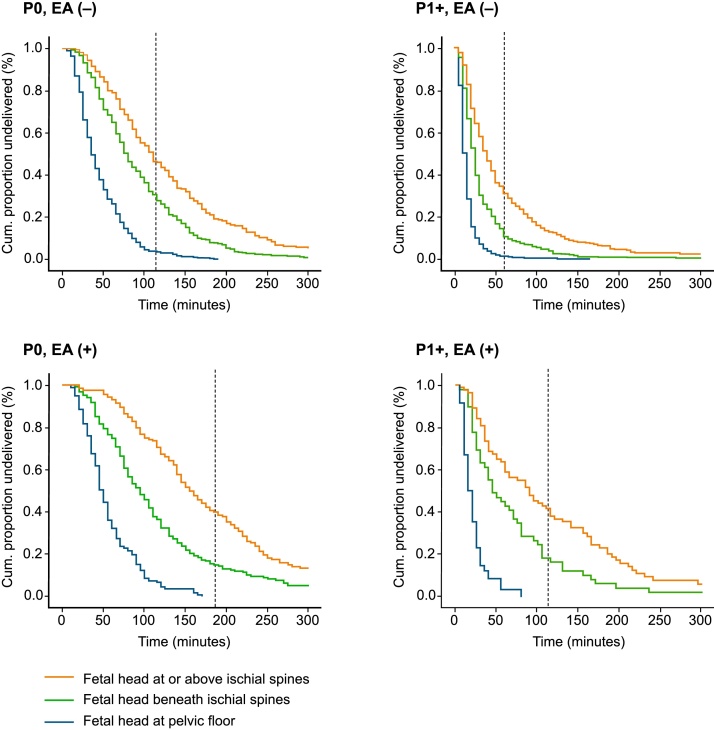
a-d Duration curves of duration of second stage of labor by station of the fetal head stratified by parity and EA use. The vertical dotted lines represent the upper limit for acceptable duration of second stage of labor defined by > 2 h without and > 3 h with epidural analgesia in nulliparous women, and > 1 h and > 2 h, respectively, in parous women. Cesarean deliveries were censored. P0, nulliparous women; P1+, parous women; EA, epidural analgesia.

Median duration of the second stage of labor in nulliparous and parous women was 71 min and 14 min, and the 90^th^ (95^th^) % were 192 (244) min and 68 (113) min, respectively. Receiving EA in the first stage of labor was associated with a longer second stage of labor, with a median (95^th^ %) duration that was was 32 (103) min and 17 (123) min longer in nulliparous and parous women, respectively. Pairwise analysis on categories of station of fetal head found significantly longer durations of second stage of labor among both nulliparous and parous women who received EA when compared to those who did not (for all percentiles, Table 2). In addition, we observed a consistent pattern of longer duration of second stage of labor by increasing distance from the pelvic floor at full cervical dilation for both parity classes and subsets of EA use. This pattern is graphically illustrated by survival curves for duration of second stage of labor (Fig. 2a-d).

**Table 2:** Duration of the second stage of labor (min) by station of the fetal head at complete cervical dilation, stratified by parity and use of epidural analgesia

Parity	Station of the fetal head at complete cervical dilation	Without EA 50^th^/ 90^th^/ 95^th 1^	With EA 50^th^/ 90^th^/ 95^th 1^
Nulliparous	At pelvic floor	34/ 85/ 99	45/ 98/ 122
	Beneath ischial spines	75/ 163/ 202	92/ 223/ 282
	At/above ischial spines	105/ 237/ 288	143/ 291/ 344
Parous	At the pelvic floor	10/ 24/ 35	14/ 38/ 54
	Beneath the ischial spines	20/ 63/ 103	42/ 164/ 212
	At/above the ischial spines	35/ 129/ 188	87/ 216/ 290

^1^Percentiles of duration.

EA, epidural analgesia.

In total, 470 women (14.2 %) had a prolonged second stage of labor; 5.3 % when the fetal head was at the pelvic floor, 36.2 % when it was beneath the ischial spines, but above the pelvic floor, and 58.5 % when the fetal head was at/above the ischial spines at complete cervical dilation. Women with prolonged second stage of labor were older, more often nulliparous and had a higher gestational age (41-42 weeks) (Table 3). In addition, they more often had prolonged first stage of labor and fetal birthweight > 4000 g. Station of the fetal head at complete cervical dilation was significantly associated with prolonged second stage of labor (Table 4).

**Table 3:** Characteristics of the sample by prolonged second stage of labor.

Variables	Not prolonged n = 2841 (%)	Prolonged n = 470 (%)	P- value
Maternal age (years) < 25 years 25-34 ≥ 35	542 (19.1) 1715 (60.4) 584 (20.6)	66 (14.0) 299 (63.6) 105 (22.3)	.03
Parity Nulliparous Parous	1100 (38.7) 1741 (61.3)	286 (60.9) 184 (39.1)	< .01
BMI (kg/ m^2^) < 18,5 18,5 -24,9 25,0-29,9 > 30,0 Missing	307 (10.8) 1588 (55.9) 563 (19.8) 350 (12.3) 33 (1.2)	39 (8.3) 289 (61.5) 97 (20.6) 39 (8.3) 6 (1.3)	.03
Gestational age (weeks) 37- 38 39- 40 41- 42	397 (14.0) 1572 (55.3) 872 (30.7)	54(11.5) 236 (50.2) 180 (38.3)	< .01
Induction of labor	591 (20.8)	101 (21.5)	.73
Prolonged 1^st^ stage of labor	225 (9.0)	80 (17.0)	<.01
Oxytocin in 1^st^ stage	440 (15.5)	90 (19.1)	.05
EA in first stage	543 (19.1)	119 (25.3)	<.01
Fetal birthweight > 4000 g	488 (17.2)	121 (25.7)	<.01
Station of fetal head at complete cervical dilation At pelvic floor Beneath ischial spines At/above ischial spines	1528 (53.8) 815 (28.7) 498 (17.5)	25 (5.3) 170 (36.2) 275 (58.5)	< .01

P-values from Chi-square tests.

BMI, body mass index; EA, epidural analgesia; BW, fetal birth weight.

^1^Duration >90^th^ %.

**Table 4:** Determinants of prolonged second stage of labor.

Variables in model	Women N	Prolonged second stage n (%)	aOR	95 % CI
Station of fetal head at cervical dilation At pelvic floor	1553	25 (1.6)	1.0	-
Beneath ischial spines	985	170 (17.3)	13.1	8.5- 20.1
At/above ischial spines	773	275 (35.6)	32.9	21.5- 50.2
Prolonged 1^st^ stage of labor	335	80 (23.9)	1.4	1.1- 1.9
Fetal birhtweight > 4000 g	609	121 (19.9)	1.5	1.2- 1.9
Maternal age (years) < 25	608	66 (10.9)	1.0	-
25- 34	2014	299 (14.9)	1.6	1.2- 2.2
≥ 35	689	105 (15.2)	1.6	1.1- 2.3

Maternal pre-pregnancy body mass index, onset of labor and gestational age had no confounding effect, thus not included in the model.

aOR, adjusted odds ratio; CI, confidence intervals

The adjusted odds ratio (aOR) for prolonged second stage of labor was 13.1 times higher when the fetal head was beneath the ischial spines, but above the pelvic floor, and 32.9 times higher when the fetal head was at/above the ischial spines compared to when the head was at the pelvic floor. Long duration of first stage of labor (> 90^th^ %), fetal birthweight > 4000 g, and maternal age independently predicted prolonged second stage of labor, whereas gestational age and onset of labor were not associated with this outcome. The strength of the association between categories of station of fetal head and prolonged second stage of labor varied less than 4% across all investigated confounders (Table 4) (no confounding). In addition, the impact of station of the fetal head on prolonged second stage of labor was evenly distributed across categories of possible predictors (no interaction).

## Comment

We found a strong association between station of the fetal head at full cervical dilation and duration of second stage of labor in both nulliparous and parous women. We observed a consistent pattern of increasing duration of second stage of labor with increasing distance from the pelvic floor for both parity classes and parity-stratified subsets of EA use. Clinically this association is reasonable, however few studies have systematically assessed and documented this relationship. This information can be helpful for health care providers when presenting expectations for labor progress during the second stage of labor, and for encouraging laboring women to endure a time-demanding delivery.

New ACOG/SMFM recommendations ^14^ from 2014 state that pushing can continue for 3 h without progress in fetal descent or rotation in nulliparous women and 2 h in multiparous women prior to diagnosing labor arrest, and this limit is extended for an additional hour when EA is provided as long as progress is documented. However, our study found that station of the fetal head had a major impact on the duration of second stage of labor. Clinical assessment of station of the fetal head when reaching the second stage of labor, and documentation of EA use, can help clinicians understand the large variations in the duration curves of the second stage of labor, especially when supplemental ultrasound examination are not available to determine station of the fetal head. [17] Furthermore, continuous fetal monitoring must confirm neonatal safety [7,18].

Where we found 91 %, Graseck et al. [19] reported that 95 % of all women had a station of the fetal head at or below the ischial spines at full cervical dilation. More than 50 years ago, Friedman [20] reported that *“the higher the station at the onset of the deceleration phase, the more protracted the labor in the deceleration phase and second stage is likely to be”.* Piper et al [21] reported that station and position of the fetal head at complete cervical dilation were two of the many factors that influence duration of second stage of labor.

Kimmich et al [22] reported that EA may decelerate fetal descent in the active phase of labor. In our study, a higher proportion of women who received EA had a recorded station of the fetal head at/above the ischial spines at the start of the second stage of labor, which could be related to a higher station when EA was administered. Women with slow labor progress may be more likely to ask for EA, and thus, by indication, be more likely to have prolonged first stage labor. Indeed, women with prolonged first stage of labor (> 90^th^%), the proportion with prolonged second stage nearly doubled, which is in agreement with Nelson et al [23] who reported that the length of the second stage of labor in nulliparous women increased significantly with increasing length of the first stage of labor.

Several retrospective studies [[4], [5], [6],^8^,^10^], have reported an association between EA use and longer duration of second stage of labor. Our estimates of the 95^th^ % duration of second stage of labor in nulliparous women who did not receive EA (3.4 h) and those who received EA (4.5 h) are very similar to what Cheng et al reported in a US study [4] comprising 42 000 women (women without EA: 3.3 h; women with EA: 5.6 h). Later Zhang et al [10] reported shorter 95^th^ % for duration of second stage of labor for both nulliparous women without EA (2.8 h) and with EA (3.6 h). In the latter study only 1/3 of the study population was eligible for duration analysis and only deliveries with normal neonatal outcomes were included. Consistent results in line with our results have been reported [1,5] for parous women and use of EA.

Duration of first stage of labor, maternal age, fetal birthweight and gestational age were independent predictors of prolonged second stage of labor without any confounding effects on the association between station of the fetal head and duration of second stage of labor. Despite adjustment analysis, residual confounding might be present, for example by rotation of the occiput posterior position of the fetal head when passing through the pelvis. We had data for position at time of delivery, and occiput posterior position at delivery (6.9%) was associated with prolonged second stage, but this variable had minimal confounding effect on the station estimates. We did not include position in the model as we lacked information on position of the fetal head through the passage of the pelvis. One study showed that two-thirds of occiput positions when reaching the ischial spines will rotate to an anterior position at the time of delivery. [24]

The strengths of our study include the long-term utilization of an established electronic medical birth record system (PARTUS), a steady study population, committed employees and validated outcome data. The extensive literature review we performed confirms that we included the most common confounders when analyzing station of the fetal head as a primary exposure for duration of second stage of labor. Limitations of our study are the retrospective study design and that we did not considered rate of descent. Further, the definitions of onset of first and second stages of labor will influence duration curves. The assessment of station of the fetal head and cervical dilation was done by vaginal exploration when indicated, not at determined intervals, thus the definition of onset of the second stage, can in some cases, be somewhat arbitrary. This subjective examination method is not very reliable and may lead to intra- and inter-observer biases [25] as demonstrated in a birth simulator study [26]. Tutschek et al [17] found that intrapartum ultrasound examinations were more reproducible in the assessment of labor progression than digital vaginal palpation. Furthermore, selection of the study sample, clinical practice and lack of standardized protocols on prolonged second stage of labor may also contribute to variations in duration estimates. These issues may affect the generalizability of our study results, but they mimic the real-world scenario in the delivery room.

We found that station of the fetal head at complete cervical dilation had a significant impact on duration of second stage of labor and on the risk of prolonged second stage of labor. Assessment of station and position of the fetal head must be considered important factors in the clinical examination of laboring women to anticipate remaining time to delivery and the likelihood of achieving vaginal delivery. Changing the guidelines for the management of the second stage of labor exclusively based on the duration of second stage, may be an oversimplification of the complex process of labor.

## Author contributions

FES designed the study. EL did data collection. EL/FES ran consistency analyses, cleaned data, and analyzed data. EL was lead author. EL/FES interpreted the results, evaluated literature, and agreed upon the final manuscript for submission.

## Funding

The study has received funding from the10.13039/501100007137Northern Norway Regional Health Authority.

## Declaration of Competing Interest

None.
